# Supplementary material for: MetaQTL: a package of new computational methods for the meta-analysis of QTL mapping experiments
Source: BMC Bioinformatics. 2007 Feb 8;8:49. doi: 10.1186/1471-2105-8-49 (PMC1808479; doi:10.1186/1471-2105-8-49)
Supplement: Additional File 7 — MetaQTL Package : jar file and tutorial. This Zip archive contains both the MetaQTL JAR file and the files of the tutorial. [file 1471-2105-8-49-S7.zip › org.inra.metaqtl/doc/org/thalia/bio/util/class-use/ILGroupPosition.html]

Uses of Interface org.thalia.bio.util.ILGroupPosition


|  |  |  |
| --- | --- | --- |
| **Overview** \| **Package** \| **Class** \| **Use** \| **Tree** \| **Deprecated** \| **Index** \| **Help** | |  |
| PREV   NEXT | **FRAMES**    **NO FRAMES**     **All Classes** |  |


---


## **Uses of Interface org.thalia.bio.util.ILGroupPosition**

| Packages that use ILGroupPosition | |
| --- | --- |
| **org.thalia.bio** |  |
| **org.thalia.bio.entity** |  |
| **org.thalia.bio.util** |  |

Uses of ILGroupPosition in org.thalia.bio

| Methods in org.thalia.bio that return ILGroupPosition | |
| --- | --- |
| `ILGroupPosition` | `IBioLocus.getPosition()` |

| Methods in org.thalia.bio with parameters of type ILGroupPosition | |
| --- | --- |
| `void` | `IBioLocus.setPosition(ILGroupPosition position)` |

Uses of ILGroupPosition in org.thalia.bio.entity

| Fields in org.thalia.bio.entity declared as ILGroupPosition | |
| --- | --- |
| `protected  ILGroupPosition` | `Locus.position` |

| Methods in org.thalia.bio.entity that return ILGroupPosition | |
| --- | --- |
| `abstract  ILGroupPosition` | `AlleleContainer.getPosition()` |
| `ILGroupPosition` | `Locus.getPosition()` |

| Methods in org.thalia.bio.entity with parameters of type ILGroupPosition | |
| --- | --- |
| `abstract  void` | `AlleleContainer.setPosition(ILGroupPosition position)` |
| `void` | `Locus.setPosition(ILGroupPosition position)` |

Uses of ILGroupPosition in org.thalia.bio.util

| Classes in org.thalia.bio.util that implement ILGroupPosition | |
| --- | --- |
| `class` | `EstimatedLocusPosition`             Class Description Here |
| `class` | `LGroupPosition`             Class Description Here |
| `class` | `StaticLocusPosition`             Class Description Here |

---


|  |  |  |
| --- | --- | --- |
| **Overview** \| **Package** \| **Class** \| **Use** \| **Tree** \| **Deprecated** \| **Index** \| **Help** | |  |
| PREV   NEXT | **FRAMES**    **NO FRAMES**     **All Classes** |  |


---
